# Frequency-Dependent Effects of Cerebellar Repetitive Transcranial Magnetic Stimulation on Visuomotor Accuracy

**DOI:** 10.3389/fnins.2022.804027

**Published:** 2022-03-18

**Authors:** Yun R. Lien, Yi-Cheng Lin, Shang-Hua N. Lin, Ching-Po Lin, Li-Hung Chang

**Affiliations:** ^1^Institute of Neuroscience, National Yang Ming Chiao Tung University, Taipei, Taiwan; ^2^Taipei Municipal Gan-Dau Hospital, Taipei, Taiwan; ^3^Education Center for Humanities and Social Sciences, National Yang Ming Chiao Tung University, Taipei, Taiwan; ^4^Institute of Philosophy of Mind and Cognition, National Yang Ming Chiao Tung University, Taipei, Taiwan

**Keywords:** cerebellum, repetitive transcranial magnetic stimulation, visuomotor coordination, visuomotor accuracy, visuomotor stability, frequency-dependent effect

## Abstract

The cerebellum plays a critical role in acquiring visuomotor skills. Visuomotor task mastery requires improving both visuomotor accuracy and stability; however, the cerebellum’s contribution to these processes remains unclear. We hypothesized that repetitive transcranial magnetic stimulation (rTMS) of the cerebellum exerts frequency-dependent modulatory effects on both accuracy and stability in subjects performing a visuomotor coordination task (i.e., pursuit rotor task). We recruited 43 healthy volunteers and randomly assigned them to the high-frequency (HF), low-frequency (LF), and sham rTMS groups. We calculated changes in performance of the pursuit rotor task at the highest rotation speed and the minimum distance from target as indices of accuracy. We also calculated the intertrial variability (standard deviations) of time on target and distance from target as indices of stability. Visuomotor accuracy was significantly enhanced in the HF group and disrupted in the LF group compared to the sham group, indicating frequency-dependent effects of rTMS. In contrast, both HF and LF rTMS demonstrated no significant change in visuomotor stability. Surprisingly, our findings demonstrated that the accuracy and stability of visuomotor performance may be differentially influenced by cerebellar rTMS. This suggests that visuomotor accuracy and stability have different underlying neural mechanisms and revealed the possibility of training strategies based on cerebellar neuromodulation.

## Introduction

The cerebellum plays an essential role in several forms of motor learning (Ito, 1984), especially when visuomotor coordination is necessary for supervised sensorimotor learning and procedural learning processes (Brown et al., 1993; Flament et al., 1996; Molinari et al., 1997; Manto et al., 2012; Knowlton et al., 2017; Raymond and Medina, 2018). For example, many motor processes involve appropriately responding to sensory-based inputs, reacting with precise timing, and performing actions that require specific positions and movements of the limbs. Specifically, the cerebellum coordinates movement by adapting motions and changing the limb trajectory in an accurate and stable manner. Accuracy refers to response in a spatially and temporally appropriate manner (Ivry et al., 1988), while stability refers to the intertrial variability in visuomotor performance (Hashimoto et al., 2015). To complete the visuomotor task, both accuracy and stability are important indicators of motor performance during learning. Therefore, it is important to explore how accuracy and stability changes in the visuomotor learning and their possible contributions to complete the visuomotor task.

Learning performance can be modulated through different kinds of non-invasive brain stimulation of relevant cortical regions (Muellbacher et al., 2001; Avanzino et al., 2015). Repetitive transcranial magnetic stimulation (rTMS) is an effective form of non-invasive neuromodulation that provides localized stimulation and modulates the excitability (and subsequent plasticity) of the cerebral cortex in a frequency-dependent manner. In particular, high-frequency (HF) rTMS increases while low-frequency (LF) rTMS decreases cortical excitability in the target region (Ziemann et al., 1996; Hallett, 2000; Maeda et al., 2000; Pascual-Leone et al., 2000; Walsh and Cowey, 2000). Regarding the rTMS frequency-dependent effects on the cerebellum, an *in vitro* study found that LF rTMS induced short-term inhibition, whereas HF rTMS induced the excitation effect with electrophysiological evidences (Tang et al., 2016). Langguth et al. (2008) demonstrated that motor cortex excitability decreased after LF cerebellar rTMS with a frequency-dependent effect, but behavioral data were lacking (Langguth et al., 2008). In another way, Wang et al. (2020) found that cerebellar activation changed only after motor cortex rTMS with HF (10 Hz) but not with LF (1 Hz) cortical stimulation (Wang et al., 2020). However, the frequency-dependent effect of cerebellar rTMS was not examined. Hence, an investigation of the frequency-dependent manipulation of cerebellar rTMS along with a behavior task may help us examine the frequency-dependent effects on cerebellar non-invasive brain stimulations and further explore the role of the cerebellum in visuomotor functions.

Multiple brain regions, such as the motor cortex, visual cortex, and sensory cortex, must work together to perform a single movement. These regions may be involved in the performance of visuomotor coordination tasks. Multiple cerebro-cerebellar networks are recruited during the learning process in visuomotor coordination tasks, including the visual cortex, attention networks, and working memory systems (Stoodley and Schmahmann, 2009). Therefore, cerebellar neuromodulation in the context of learning in a visuomotor coordination task may have an effect on multiple regions of the cerebral cortex by altering these cerebro-cerebellar connections (Koch, 2020; Spampinato and Celnik, 2021). By measuring the activity of the visual and motor cortex, we aimed to investigate whether neuromodulation on the cerebellum affects the excitability of the cortical areas which might associate with visuomotor tasks.

In the present study, we sought to determine how cerebellar rTMS influences both accuracy and stability of performance in a visuomotor coordination task, namely, the pursuit rotor task (Grafton et al., 1994; Gupta et al., 2017; Knowlton et al., 2017). To examine potential changes in cortical regions mediated through cerebro-cerebellar connections, we assessed the reactivity of the visual and motor cortices after rTMS and behavioral training. We hypothesized that cerebellar rTMS can modulate both behavioral accuracy and stability in a frequency-dependent manner; in particular, we hypothesized that HF rTMS would increase the excitability of the cerebellum and improve visuomotor accuracy and stability, whereas LF rTMS would inhibit cerebellar function and decrease visuomotor accuracy and stability.

## Materials and Methods

### Participants

Forty-three healthy volunteers with no history of neurological or vision problems were randomly assigned to three groups—sham (*N* = 12; age = 23.5 ± 2.5 years; 6 men, 6 women), LF (*N* = 15; age = 22.1 ± 1.3 years; 6 men, 9 women), and HF (*N* = 16; age = 22.8 ± 1.4 years; 6 men, 10 women)—to receive different rTMS interventions. All of the participants gave informed consent and obtained neuropsychiatric assessments, including the Wechsler Adult Intelligence Scale (WAIS)-III (digit–symbol coding and letter–number sequencing) and useful field of vision (UFOV, a standardized visual attention task (Ball et al., 1988; Chang et al., 2014), before the experiment started. All the experiments were conducted at the National Yang Ming Chiao Tung University. We found no significant differences among the three groups in age, gender ratio, years of education, or scores on neuropsychiatric assessments. This study was approved by the Institutional Review Board of the National Yang Ming Chiao Tung University.

### Experimental Procedure

**FIGURE 1:**
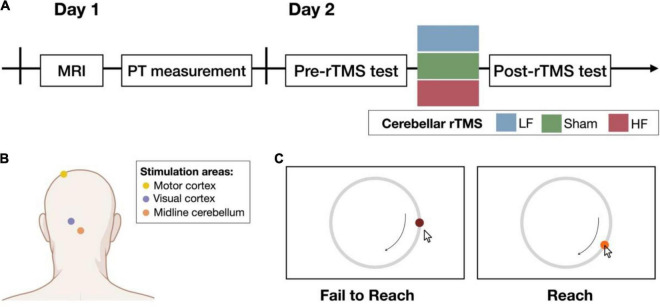
Experimental procedure. **(A)** On day 1, structural imaging was performed before the experiment began. The phosphene threshold (PT) was measured and used as the reference output intensity for cerebellar rTMS. On day 2, participants were randomly assigned to one of the three groups: low-frequency (LF), sham, or high-frequency (HF) group. Performance on the behavioral task was assessed before and after the cerebellar rTMS intervention. The control experiments followed the same procedure on day 2 in the following 2 weeks. **(B)** The yellow and purple dots illustrate the target site of the resting motor threshold (rMT) and PT on the motor and visual cortex, respectively. The orange dot illustrates the midline cerebellum, the target of interest for the cerebellar rTMS intervention (created with BioRender.com). **(C)** Participants were asked to follow a target that moves on a circular track. The color of the target was presented as the feedback about task success (right) or failure (left). If the participant did not reach the target, the target would not change color. Once the participant reached the target, the target turned light red, and participants could learn whether they reached the target to immediately improve their behavioral performance.

On the first day of the experiment, the participants underwent T1-weighted magnetic resonance imaging (MRI; 3T Siemens Magnetom Tim Trio, Germany) to determine the regions of interest in the cerebellum and visual cortex. After the MRI session, the phosphene threshold (PT) was determined to provide a reference value for the intensity of the rTMS that was subsequently used (the procedure is described in more detail below). On the second day, the participants performed the behavioral task (20–30 min/session) before and after the rTMS intervention (within 10 min/session; see Figure 1A). In particular, the rTMS intervention and the following post-test would take about 40 min.

### Transcranial Magnetic Stimulation Paradigm

TMS was performed using a Magstim Rapid^2^ stimulator (Magstim, United Kingdom) with an air-cooled figure-of-eight coil in the LF and HF groups or an uncoated D70 Alpha Sham Coil in the sham group. rTMS was performed on the midline cerebellum (see Figure 1B; Théoret et al., 2001; Argyropoulos and Muggleton, 2013; Gupta et al., 2017; Summers et al., 2018) with an output intensity of 100% PT (Brückner and Kammer, 2014, 2015). The LF group received 600 pulses at 1 Hz, and the HF group received 600 pulses at 10 Hz (Langguth et al., 2008; Vasant et al., 2015). To avoid noticing which rTMS intervention they received, a single-blind design was applied while performing the entire experiment. All participants were instructed to wear eye masks before starting the rTMS session. Additionally, the background noise was also controlled in all groups. The stimulation parameters of the sham group were the same as those of the HF group but used a different coil. For the markers to choose the stimulation site, before T1 scanning, five markers were placed near the inion, including Iz, Oz, O1, O2, and 1 cm below Iz using the 10–20 system. The T1 images helped us determine the more accurate site on the scalp by estimating the distance between the target site and the markers. The approximate cerebellar target site is localized 1–2 cm bellow the inion.

### Phosphene Threshold and Motor Threshold Measurements (Control Study)

To determine whether the observed behavioral changes were associated with altered visual or motor cortical regions reactivity, both the PT and resting motor threshold (rMT) were measured immediately before and after the cerebellar rTMS intervention (see Figure 1B). In this control study, the 66 (sham: *N* = 11; LF: *N* = 25; HF: *N* = 30) and 44 (sham: *N* = 14; LF: *N* = 18; HF: *N* = 12) volunteers (see Supplementary Table 1) were recruited for the PT and motor measurement study, respectively, and assigned to the three groups for different cerebellar rTMS intervention (sham, LF, and HF).

Both PT and rMT were assessed with an adaptive parameter estimation by sequential testing (PEST) procedure (adaptive PEST for TMS),^1^ which estimates the cortical threshold with a non-parametric algorithm. For PT measurement, the participants opened their eyes in a dark room and reported the presence of phosphenes in their visual fields after single-pulse TMS was delivered to the visual cortex (Marg and Rudiak, 1994). For rMT measurement, electrode patches were placed on the abductor pollicis brevis muscle (APB) of the dominant hand, and a TMS coil was placed on the APB hotspot in the contralateral hemisphere (Wilson et al., 1993). The motor hotspot was determined by continuous 10-trial of single-pulse TMS with 75% output intensity, which successfully evoked the motor-evoked potential (MEP). The successful trial was considered if the peak-to-peak amplitude ≥ 50 μV. Once the location of the motor hotspot was identified, the TMS coil was fixed in place for the estimation of rMT. The rMT was determined by adjusting the intensity output of TMS, which can evoke the minimum MEP with the PEST (Herbsman et al., 2009).

### Behavioral Tasks

The behavioral tasks, i.e., the pursuit rotor task and the Mackworth clock test, were performed on a desktop computer running Windows 7 and were programmed with Psychology Experiment Building Language 2.0 (PEBL) (Mueller and Piper, 2013).

#### Pursuit Rotor Task

Before (pre-test) and after (post-test) the cerebellar rTMS intervention, the participants performed the pursuit rotor task, a widely used procedural learning task that can examine both visuomotor tracking and hand–eye coordination abilities (Knowlton et al., 2017). For this task, the participants were asked to follow a target (diameter = 1.3 cm; visual angle = 1.66°) moving on a circular track (diameter = 13 cm; visual angle = 16.44°) with a drawing pen and tablet (WACOM Intuos; active area: 152 × 95 mm). If they successfully reached the target, the dark red target turned light red as a feedback signal (see Figure 1C). Performance at the following eight levels with different rotation speeds was assessed: 0.13, 0.16, 0.23, 0.3, 0.4, 0.5, 0.6, and 0.7 rotations per second (rps). The first level was used for practice, and the other seven levels were used for testing. Six trials were performed at each testing level, and each trial lasted 15 s. During each session (pre-test and post-test), the participants moved from lower rotation speeds to higher rotation speeds, stopping at the level at which their maximum time on target was below 7.5 s (i.e., less than 50% successful tracking). Before the task session started, the participants were encouraged to do their best to achieve the best performance to enter the next level.

#### Mackworth Clock Test (Control Task)

To determine whether cerebellar modulation altered attention (also affected the performance by modulated attention system), the participants performed the Mackworth clock test, which tested long-term vigilance while detecting signals (Mackworth, 1948). Participants were instructed to press the space key on the keyboard if the target skipped a position along the circular track. The probability of a “skip” trial was 0.4. Practice level consisted of 60 trials, while the test level contained 120 trials. The pre-test consisted of a practice level and a test level, and the post-test included a test level.

### Data Analysis

#### Pursuit Rotor Task

To characterize performance in the pursuit rotor task, two scores were used—distance from target and time on target. The distance from target was defined as the mean distance between the cursor and the target (Grafton et al., 2001). Time on target was defined as the total time that participants kept the cursor in the light red target on the circle (Piper, 2011). To determine the appropriate speed to compare the performance changes (i.e., from before to after the rTMS intervention) for each participant, we fitted logistic curves to their performance with the maximum and average time on target at each speed level, respectively. From these curves, the estimated speed at which the participant could reach the target with threshold levels (50, 55, 60, 65, 70, and 75%) was used to examine the spatial aspect of visuomotor learning. With different rotation speeds, logistic curves derived from the minimum distance and mean distance from target displayed the accurate visuomotor learning performance.

The trial with the maximum time on target in each level and the trial with the minimum distance from target in each level, as well as the mean value in each level, were transformed into logistic curves by individual psychometric functions. From these curves, the rotation speeds at which 50, 55, 60, 65, 70, and 75% of the total time were spent on the target and the estimated distance from the target corresponding to the rotation speeds were selected as indicators of participant performance. The threshold levels range from 50 to 75% of tracking accuracy was determined because all participants could achieve it and hence fit better in the psychometric curve. To examine the performance of motor learning acquisition, the area under the curve (AUC) within that threshold range was calculated and regarded as the indicator of the continuous measurement of learning acquisition (see Supplementary Figure 1).

To assess the rotation speed and mean distance change in relation to performance accuracy, we examined behavior performance change across the factors cerebellar rTMS groups (HF, LF, and sham) and sessions (pre- vs. post-rTMS intervention) by two-way mixed ANOVA.

Furthermore, the behavioral improvement for the distance from target was estimated using formula (1), and the improvement for the rotation speed was calculated with formula (2).


(1) $${\text{Improvement}}_{\mathrm{distance}\mathrm{from}\mathrm{target}}=\frac{-\left({\text{AUC}}_{\mathrm{Posttest}}-{\text{AUC}}_{\mathrm{Pretest}}\right)}{{\text{AUC}}_{\mathrm{Pretest}}}\times 100\%$$

(2) $${\text{Improvement}}_{\mathrm{rotation}\mathrm{speed}}=\frac{\left({\text{AUC}}_{\mathrm{Posttest}}-{\text{AUC}}_{\mathrm{Pretest}}\right)}{{\text{AUC}}_{\mathrm{Pretest}}}\times 100\%$$

We assessed and compared accuracy improvements, an indicator of learning, between the cerebellar rTMS groups HF, LF, and sham by one-way ANOVA with the factors group (HF, LF, and sham). If there was a significant main effect of group or an interaction of session and group, pairwise *t*-tests with Bonferroni correction were performed for *post hoc* analyses.

Furthermore, to compare performance stability, another indicator of learning, we evaluated the intertrial variability in time on target and distance from target in the pursuit rotor task. The intertrial variability was defined by the sum of within-level standard deviation (SD) in each level of rotation speed (0.16, 0.23, 0.3, and 0.4 rps; each level had six trials). Improvement in intertrial variability was estimated using formula (3).


(3) $${\text{Improvement}}_{\mathrm{intertrial}\mathrm{variability}}=\frac{-\left({\text{SD}}_{\mathrm{Posttest}}-{\text{SD}}_{\mathrm{Posttest}}\right)}{{\text{SD}}_{\mathrm{Pretest}}}\times 100\%$$

All of the statistical analyses were conducted using the R package, “rstatix” (Kassambara, 2020). All statistical results were corrected for with Bonferroni correction. The effect sizes were reported as partial eta squared (η^2^*_*p*_*) values for *F*-test. The interpretation of coefficient η^2^*_*p*_* is as follows: η^2^*_*p*_* = 0.01 indicates a small effect size; η^2^*_*p*_* = 0.06 indicates a medium effect size; η^2^*_*p*_* = 0.14 or higher indicates a large effect size.

#### Mackworth Clock Test

The performance in the Mackworth clock test was expressed as the d-prime score generated by the R package “psycho” (Makowski, 2018) and the mean reaction time on the correct trials. Both variables were assessed by two-way mixed ANOVA with the factors group (HF, LF, and sham) and session (pre- vs. post-rTMS intervention). Significant main effects were examined by *post hoc* pairwise *t*-tests with Bonferroni correction.

#### Phosphene Threshold and Resting Motor Threshold

To determine whether cerebellar rTMS interventions would affect the related cortical connections in visual or motor functions, both PT and rMT were assessed by two-way mixed ANOVA across the three rTMS groups. *Post hoc* analyses were performed using pairwise *t*-tests, and the results were corrected by Bonferroni correction.

## Results

### Accuracy of Performance in the Pursuit Rotor Task

**FIGURE 2:**
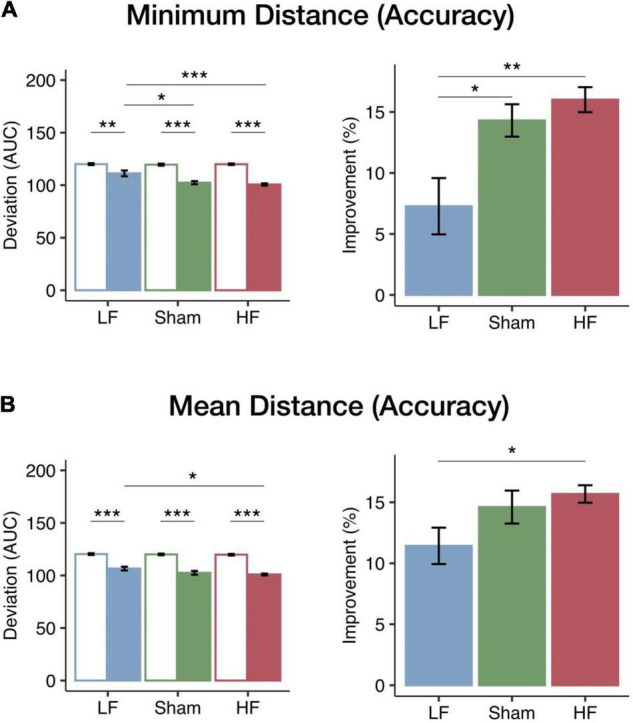
Changes in visuomotor accuracy in the minimum and mean distances from target among the three rTMS groups. The bars (blue, LF, *N* = 15; green, sham, *N* = 12; red, HF, *N* = 16) represent the mean AUC of deviation (left panel) in the pre-test (empty bar) and post-test (solid bar) and mean improvements (right panel). **(A)** The performance and improvements in the minimum distance from target. All groups had significant decreases in the minimum distance after intervention. Furthermore, in the post-test, both the HF and the sham groups had a significantly smaller deviation than the LF group. The improvements of the HF and sham groups were significantly greater than those of the LF group. **(B)** Performance and improvements in the mean distance from target. All groups had significant decreases in mean distance after intervention; moreover, the HF group had not only a significantly smaller deviation but also a greater improvement than the LF group. Error bars indicate the standard error of the mean. Bonferroni correction was applied to all *p*-values (*p < 0.05; **p < 0.01; ***p < 0.001).

To assess the impact of HF and LF cerebellar rTMS on visuomotor performance accuracy, we calculated the AUC of the minimum and mean distances from target performance curves at the speed at which participants achieved threshold levels from 50 to 75% of tracking accuracy in the pursuit rotor task. The AUC of the minimum distance represented the best performance accuracy, and the AUC of the mean distance represented the mean performance accuracy. The baseline performance of the three groups did not differ. Two-way mixed ANOVA tests revealed significant main effects of session (pre-test and post-test) on both minimum distance [*F*_(1_, _40)_ = 157.87, *p* < 0.001, η^2^*_*p*_* = 0.80] and mean distance from target [*F*_(1_, _40)_ = 371.70, *p* < 0.001, η^2^*_*p*_* = 0.90]. Specifically, there was an overall decrease in the distance from target post-rTMS intervention compared to baseline. The significant main effects of the rTMS group were found on the minimum distance from target [*F*_(2_, _40)_ = 7.43, *p* = 0.002, η^2^*_*p*_* = 0.27] but not on the mean distance from target [*F*_(2_, _40)_ = 2.96, *p* = 0.063, η^2^*_*p*_* = 0.13]. There were also significant interactions on both the minimum [*F*_(2_, _40)_ = 7.61, *p* = 0.002, η^2^*_*p*_* = 0.28] and mean distances from target [*F*_(_*_2_*, _40)_ = 3.23, *p* < 0.05, η^2^*_*p*_* = 0.14], suggesting that different cerebellar rTMS have distinct effects on visuomotor learning. *Post hoc* analysis of minimum distance from target demonstrated that the deviation in the post-test of the LF group was significantly larger than that of the HF (*p* < 0.001) and sham (*p* = 0.010) groups (see Figure 2A). On the other hand, the *post hoc* analysis of the mean distance from target demonstrated that the deviation in the post-test of the LF group was significantly larger than that of the HF group (*p* = 0.029; see Figure 2B).

The performance change was used to compare the learning efficacy of the three cerebellar rTMS groups. One-way ANOVA tests revealed significant main effects of the rTMS group on both minimum [*F*_(2_, _40)_ = 8.07, *p* = 0.001, η^2^*_*p*_* = 0.29] and mean [*F*_(2_, _40)_ = 3.56, *p* = 0.038, η^2^*_*p*_* = 0.15] distances from target. Given the significant main effects of the rTMS group, we further examined how the changes in the minimum and mean distances were modulated by the different cerebellar rTMS protocols. The improvements of the HF group were significantly larger than those of the LF group for both minimum (*p* = 0.001; see Figure 2A) and mean distances (*p* = 0.040; see Figure 2B) according to the *post hoc* analyses. Furthermore, the improvements in the minimum distance from target in the LF group were significantly less than those in the sham group (*p* = 0.020). In both aspects of the pursuit rotor task, HF cerebellar stimulation resulted in greater improvements than sham or LF stimulation, thereby showing a frequency-dependent modulation of performance accuracy in the visuomotor coordination task.

We also calculated the AUC of the highest and mean rotation speed fitting curve in the threshold levels from 50 to 75% to be the other index for performance accuracy. Regarding the highest rotation speed, mixed ANOVA showed a significant main effect of session [*F*_(_*_1_*, _40)_ = 123.96, *p* < 0.001, η^2^*_*p*_* = 0.76] and a significant interaction between session and the rTMS group [*F*_(2_, _40)_ = 5.02, *p* = 0.011, η^2^*_*p*_* = 0.20]. In the post-test, the *post hoc* analysis showed a significant increase compared to that in the pre-test in all three groups (see Supplementary Figure 2A). Furthermore, one-way ANOVA also revealed a significant main effect of rTMS groups on the improvement of rotation speed [*F*_(2_, _40)_ = 6.00, *p* = 0.005, η^2^*_*p*_* = 0.23], and the *post hoc* analysis showed that the improvements in the HF group were significantly larger than those in the LF group (*p* = 0.005). However, for the mean rotation speed, although a significant main effect of the session was found [*F*_(1_, _40)_ = 267.90, *p* < 0.001, η^2^*_*p*_* = 0.87], neither a main effect of the group [*F*_(2_, _40)_ = 0.55, *p* = 0.584, η^2^*_*p*_* = 0.03] nor an interaction between these two factors [*F*_(2_, _40)_ = 2.19, *p* = 0.125, η^2^*_*p*_* = 0.01] was observed (see Supplementary Figure 2B). The detail of the comparison of distance from target and rotation speed in three groups is reported in Supplementary Figure 3.

### Stability of Performance in the Pursuit Rotor Task: Intertrial Variability

**FIGURE 3:**
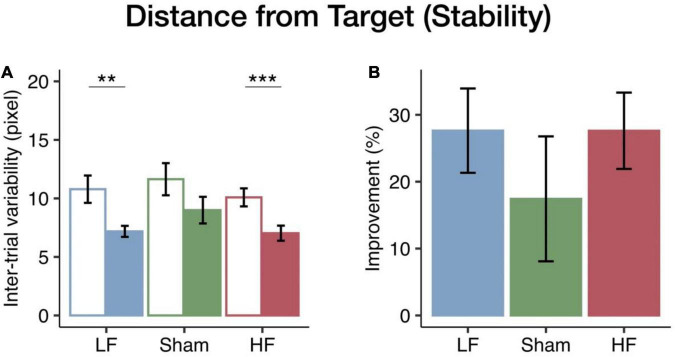
Changes in visuomotor stability in the distance from target among the three rTMS groups. **(A)** The bars (blue, LF; green, sham; red, HF) represent the mean intertrial variability in the pre-test (empty bar) and post-test (solid bar). Both the HF and LF groups had greater decreases in intertrial variability than the sham group. **(B)** The bars represent the mean improvements in the intertrial variability. Error bars indicate the standard error of the mean. Bonferroni correction was applied to all *p*-values (**p < 0.01; ***p < 0.001).

To assess the impact of HF and LF cerebellar rTMS on visuomotor performance stability, intertrial variability between trials in time on target and distance from target in the pursuit rotor task was calculated as the SD between the trials at each level. The total intertrial variability of the first four levels was considered the index of performance stability. Mixed ANOVA tests showed significant main effects of the session on the distance from target [*F*_(1_, _40)_ = 28.02, *p* < 0.001, η^2^*_*p*_* = 0.41] and time on target [*F*_(1_, _40)_ = 53.54, *p* < 0.001, η^2^*_*p*_* = 0.57]. However, there were no significant group effects in terms of distance from target [*F*_(2_, _40)_ = 1.27, *p* = 0.293, η^2^*_*p*_* = 0.06] or time on target [*F*_(2_, _40)_ = 0.25, *p* = 0.779, η^2^*_*p*_* = 0.01] and no interaction in distance from target [*F*_(2_, _40)_ = 0.22, *p* = 0.807, η^2^*_*p*_* = 0.01] or time on target [*F*_(2_, _40)_ = 0.27, *p* = 0.769, η^2^*_*p*_* = 0.01]. In the HF and LF groups, the intertrial variability in distance from target significantly decreased after cerebellar rTMS intervention (HF: *p* < 0.001; LF: *p* = 0.003; sham: *p* = 0.087) (see Figure 3A). Furthermore, the results of the time on target were similar to those of the distance from target, which also showed a significant decrease after intervention in both the HF (*p* < 0.001) and LF (*p* < 0.001) groups, in addition to the fact that there was also a significant decrease in the sham group (*p* = 0.002; see Supplementary Figure 4A).

To further investigate changes in stability among the HF, LF, and sham groups, we performed one-way ANOVA across the rTMS groups. There was no significant rTMS group effect on either time on target [*F*_(2_, _40)_ = 0.16, *p* = 0.854, η^2^*_*p*_* = 0.01; see Figure 3B] or distance from target [*F*_(2_, _40)_ = 0.65, *p* = 0.528, η^2^*_*p*_* = 0.03; see Supplementary Figure 4B]. However, both the HF and LF groups had greater improvements in performance stability than the sham group (see Figure 3B and Supplementary Figure 4B). Unlike performance accuracy, intertrial variability tended to show similar rTMS effects at both frequencies.

### Lack of Cerebellar Repetitive Transcranial Magnetic Stimulation Effects on Mackworth Clock Test Performance

To determine whether the cerebellar rTMS intervention would affect the other cognitive domains, the Mackworth clock test was performed before and after cerebellar rTMS. Baseline performance on the Mackworth clock test was not different among the three cerebellar rTMS groups. Two-way mixed ANOVA showed that there was no significant change in the d-prime score based on the rTMS group or session [group: *F*_(2_, _38)_ = 0.58, *p* = 0.566, η^2^*_*p*_* = 0.03; session: *F*_(1_, _38)_ = 0.48, *p* = 0.492, η^2^*_*p*_* = 0.01]. On the other hand, the mean reaction time in the correct trials showed a session effect [*F*_(1_, _38)_ = 7.90, *p* = 0.008, η^2^*_*p*_* = 0.17] but no rTMS group effect [*F*_(2_, _38)_ = 0.36, *p* = 0.701, η^2^*_*p*_* = 0.02]. However, *post hoc* analysis did not show any significant difference in mean reaction time. In summary, there were no significant changes in accuracy on the Mackworth clock test after rTMS, suggesting that the attention system is not affected by cerebellar rTMS intervention.

### Lack of Cerebellar Repetitive Transcranial Magnetic Stimulation Effects on Phosphene and Resting Motor Thresholds

To assess the influence of cerebellar rTMS interventions on the reactivity of relevant cortical regions, PT and rMT were determined before and after the cerebellar rTMS intervention. Two-way mixed ANOVA for PT found no significant effect based on the rTMS group [*F*_(2_, _63)_ = 0.02, *p* = 0.981, η^2^*_*p*_* = 0.00] or session [*F*_(1_, _63)_ = 1.16, *p* = 0.286, η^2^*_*p*_* = 0.02]. Furthermore, there was no significant interaction effect [*F*_(2_, _63)_ = 0.33, *p* = 0.718, η^2^*_*p*_* = 0.01]. Similarly, rMT did not show significant effects based on the rTMS group [*F*_(2_, _41)_ = 0.30, *p* = 0.746, η^2^*_*p*_* = 0.01] or session [*F*_(1_, _41)_ = 0.11, *p* = 0.743, η^2^*_*p*_* = 0.00]. The interaction between two factors in rMT also did not show a significant effect [*F*_(2_, _41)_ = 0.04, *p* = 0.964, η^2^*_*p*_* = 0.00]. In summary, there were no significant changes in PT or rMT after rTMS, indicating that cortical excitability was not affected by midline cerebellar rTMS.

## Discussion

In our study, we tested whether cerebellar rTMS could regulate visuomotor performance. We hypothesized that cerebellar rTMS would modulate both the accuracy and stability of visuomotor coordination in a frequency-dependent manner. Our results showed that cerebellar rTMS modulated only performance accuracy in a frequency-dependent manner. On the other hand, intertrial variability, an indicator of visuomotor stability, showed a trend-level effect of rTMS with potential improvements from both HF and LF stimulation. In addition, there was no evidence regarding an influence of cerebellar rTMS on the cortical networks underlying visuomotor learning.

### Frequency-Dependent Effects Were Found Only on Improvements in Visuomotor Accuracy

In our study, we showed that the improvements in the HF group were significantly greater than those in the LF group. We also found that the improvement of the best performance accuracy in the sham group was significantly larger than those in the LF group. These results supported our hypothesis that cerebellar rTMS can modulate visuomotor learning in a frequency-dependent manner, indicating that HF cerebellar rTMS enhanced learning and LF cerebellar rTMS undermined learning. Our findings were consistent with previous studies that examined the effects of cerebellar rTMS on cognitive functions, including language processing, emotion regulation (Schutter and Honk, 2008; Lesage et al., 2012), and motor learning (Koch et al., 2019). Additionally, a previous study showed that 1 Hz cerebellar rTMS in the posterior midline cerebellum impaired saccade adaptation (Jenkinson and Miall, 2010). Continuous theta-burst stimulation (cTBS), which is a form of inhibitory TMS, interfered with the processing of sensory-motor information during saccades (Colnaghi et al., 2011). In contrast, intermittent TBS (iTBS), a form of excitatory cerebellar TMS, was found to improve visuomotor learning ability in a visuomotor adaptation task (Koch et al., 2019). Furthermore, physiological evidence has demonstrated that cerebellar rTMS has frequency-dependent effects in the cerebellar cortex (Tang et al., 2016), including altered firing rates of cerebellar neurons (Tang et al., 2016) and synapse plasticity (Koch, 2010).

Although we found that the improvement in performance accuracy in the HF group was larger than that in the LF group, the improvement in the HF group was not significantly greater than that in the sham group. Our results were not fully consistent with the results of previous studies’ findings that HF rTMS resulted in better performance on behavioral tasks than sham. Our findings also showed that all groups had significantly improved performance accuracy during their post-test. These results suggest that the pursuit rotor task could be quickly learned in healthy young participants, and the practice effect of it should be taken into consideration. Therefore, it is possible that the effect of learning was strong and reduced the effect of rTMS in statistical results when comparing the HF group to the sham group. Moreover, although the effect of learning was strong, a clear inhibition of practice-based learning by LF rTMS in the minimum distance from target was observed, supporting the hypothesis about the frequency-dependent effect of cerebellar rTMS on improvements in visuomotor accuracy.

Although several studies have shown that cerebellar rTMS regulates functional networks and neural substrates throughout the brain (Koch, 2010; Rastogi et al., 2017), an understanding of the neural mechanisms underlying the effects of cerebellar rTMS interventions in modulating behavioral performance requires further investigation. Here, we found that the frequency-dependent effects of rTMS had an impact on the accuracy of behavioral performance in a visuomotor coordination task.

### The Difference Between the Accuracy and Stability of Performance in Learning a Visuomotor Coordination Task

Both accuracy and stability are important for the coordination function of the cerebellum (Ryu and Buchanan, 2012). Previous motor studies considered motor stability as one of the factors that influence motor learning abilities (Srinivasan and Mathiassen, 2012), and the temporal structure of motor variability might predict motor learning ability (Wu et al., 2014). In our study, rTMS had frequency-dependent effects on accuracy and not stability; there was a trend toward enhanced stability measured by variability in distance from the target at both frequencies, but without significance. These results were unexpected given our hypothesis that both accuracy and stability learning would be modulated by cerebellar rTMS in a frequency-dependent manner. Although the causes of these interesting differences between accuracy and stability are unknown, this differential modulation may indicate that these processes engage different mechanisms or pathways.

### The Circuits Related to Visuomotor Learning

For decades, the functions of the cerebellum have been thought to be limited to motor control and body balance. However, accumulating evidence has demonstrated that the cerebro-cerebellar connections participate in multiple cognitive functions, including attention, working memory, and emotion regulation (Bernard et al., 2012; Rastogi et al., 2017; Brissenden et al., 2018). Visual and motor cortices, and perhaps attention networks, might be involved during the learning of visuomotor coordination. In our study, we found that PT and MT, indices of visual and motor cortex excitability, did not change after either inhibitory or excitatory cerebellar rTMS interventions. Our results suggest that the effect of cerebellar rTMS on visuomotor performance might not be caused by the change of visual or motor-related connections. Due to the limitation of the experiment design, several potential biases were not fully addressed, for instance, the relation between the effect of rTMS and stimulation intensity or with motor baseline excitability. These issues should be considered in future research.

Previous studies examining the relationship between non-invasive cerebellar stimulation and cortical activation have been inconsistent. In the motor cortex, Oliveri et al. (2004) found that motor-evoked potentials (MEP) were not changed by 1 Hz cerebellar rTMS, which is similar to our results. Other studies demonstrated that motor excitability decreased after an inhibitory cerebellar rTMS intervention (Fierro et al., 2007; Li Voti et al., 2014). Li Voti et al. (2014) also showed changes in MEP after delivering cTBS to the lateral cerebellum and suggested that cerebellar cTBS contributed exogenous alterations in motor plasticity. In the visual cortex, visual mapping of the cerebellum has demonstrated that the upper vermis is involved in visual processing (Van Es et al., 2019), and this region contributes to visual working memory and saccades (King et al., 2019). Furthermore, Farzan et al. (2016) found that network-targeted cerebellar iTBS at the midline enhanced sustained and transient attentional control. However, we did not find significant differences after cerebellar rTMS, which may indicate more involvement of the visuomotor function than attention network. This difference might be caused by the fact that we only assess vigilance on detecting the signal without examining the other attention domains. Due to the limitations of the experiment design, we could not completely rule out the possibility that cerebellar rTMS could affect various aspects of attention ability. Therefore, further research is needed to clarify these inconsistent results involving the cerebellum, the cortex, and their connections.

## Conclusion

In summary, we found that cerebellar rTMS successfully modulated the accuracy of visuomotor coordination in a frequency-dependent manner. Furthermore, the stability of learning performance tended to show an enhancement by rTMS at both frequencies. We also found that the excitability of associated visuomotor cortical regions was not enhanced by cerebellar interventions in our study, although the possibility of functional connectivity changes between the cerebellum and cortical regions cannot be excluded. The cerebellum has been reported in many diseases such as spinocerebellar ataxias, Alzheimer’s disease, Parkinson’s disease, and psychiatric disorders (Pedroso et al., 2013; Castellazzi et al., 2014; Stefanescu et al., 2015; Guell et al., 2020), and compensatory phenomena were observed in chronic cerebellar-related chronic neurodegenerative diseases (Lin et al., 2020). Our study implies the possibility of visuomotor coordination training strategies that may be important for and enhance learning abilities and improve visuomotor functions in patients with cerebellum disorders.

## Data Availability Statement

The raw data supporting the conclusions of this article will be made available by the authors, without undue reservation.

## Ethics Statement

The studies involving human participants were reviewed and approved by the Institutional Review Board of National Yang Ming Chiao Tung University. The patients/participants provided their written informed consent to participate in this study.

## Author Contributions

YL, S-HL, C-PL, and L-HC designed the experiments. YL, S-HL, and L-HC performed the behavioral and TMS experiments. YL, Y-CL, S-HL, and L-HC analyzed the data. YL, Y-CL, S-HL, C-PL, and L-HC wrote the manuscript. All authors contributed to the article and approved the submitted version.

## Conflict of Interest

The authors declare that the research was conducted in the absence of any commercial or financial relationships that could be construed as a potential conflict of interest.

## Publisher’s Note

All claims expressed in this article are solely those of the authors and do not necessarily represent those of their affiliated organizations, or those of the publisher, the editors and the reviewers. Any product that may be evaluated in this article, or claim that may be made by its manufacturer, is not guaranteed or endorsed by the publisher.

## Footnotes

1
https://www.clinicalresearcher.org
