# Supplementary material for: Frequency-Dependent Effects of Cerebellar Repetitive Transcranial Magnetic Stimulation on Visuomotor Accuracy
Source: Front Neurosci. 2022 Mar 18;16:804027. doi: 10.3389/fnins.2022.804027 (PMC8971901; doi:10.3389/fnins.2022.804027)
Supplement: Supplementary file 1 [file Table_1.docx]

**­Frequency-dependent effects of cerebellar rTMS on visuomotor accuracy**

Supplementary Material

# 1.1 Supplementary Tables

|  |  | LF | Sham | HF | p-value |
| --- | --- | --- | --- | --- | --- |
| **Pursuit Rotor Task** | | | | | |
|  | N | 15 | 12 | 16 |  |
|  | Age | 22.1 (1.3) | 23.5 (2.5) | 22.8 (1.4) | p = 0.118 |
|  | Gender (M:F) | 6:9 | 6:6 | 6:10 | p = 0.856 |
| **Phosphene Threshold** | | | | | |
| All | N | 25 | 11 | 30 |  |
|  | Age | 23.3(3.3) | 22.2(1.7) | 23.0(2.0) | p = 0.250 |
|  | Gender (M:F) | 10:15 | 5:6 | 12:18 | p = 1.000 |
| Subgroup1 | N | 9 | 5 | 14 |  |
|  | Age | 21.9(1.3) | 23.4(1.9) | 22.9(1.5) |  |
|  | Gender (M:F) | 3:6 | 2:3 | 5:9 |  |
| Subgroup2 | N | 16 | 6 | 16 |  |
|  | Age | 22.3(2.0) | 23.2(4.4) | 23.1(2.4) |  |
|  | Gender (M:F) | 9:7 | 3:3 | 9:7 |  |
| **Motor Threshold** | | | | | |
| All | N | 18 | 14 | 12 |  |
|  | Age | 22.4(1.8) | 22.1(1.4) | 22.9(1.9) | p = 0.519 |
|  | Gender (M:F) | 7:11 | 5:9 | 6:6 | p = 0.796 |
| Subgroup1 | N | 8 | 4 | 7 |  |
|  | Age | 22.3(1.3) | 22.0(1.3) | 23.6(1.8) |  |
|  | Gender (M:F) | 3:5 | 2:2 | 4:3 |  |
| Subgroup2 | N | 10 | 10 | 5 |  |
|  | Age | 22.7(2.2) | 22.1(1.5) | 22.0(1.9) |  |
|  | Gender (M:F) | 4:6 | 3:7 | 2:3 |  |

**Supplementary Table 1.** Participants in the subgroup1 also took parts in the main experiment of this study (pursuit rotor task). Each measurement was performed at intervals of at least one week and led to washout of the previous cerebellar rTMS. In addition to that, subgroup1 and subgroup 2 underwent the same procedure during phosphene and motor threshold measurements, thus we combined those subgroups for the analysis of the changes of phosphene and motor thresholds after cerebellar rTMS.

## 1.2 Supplementary Figures


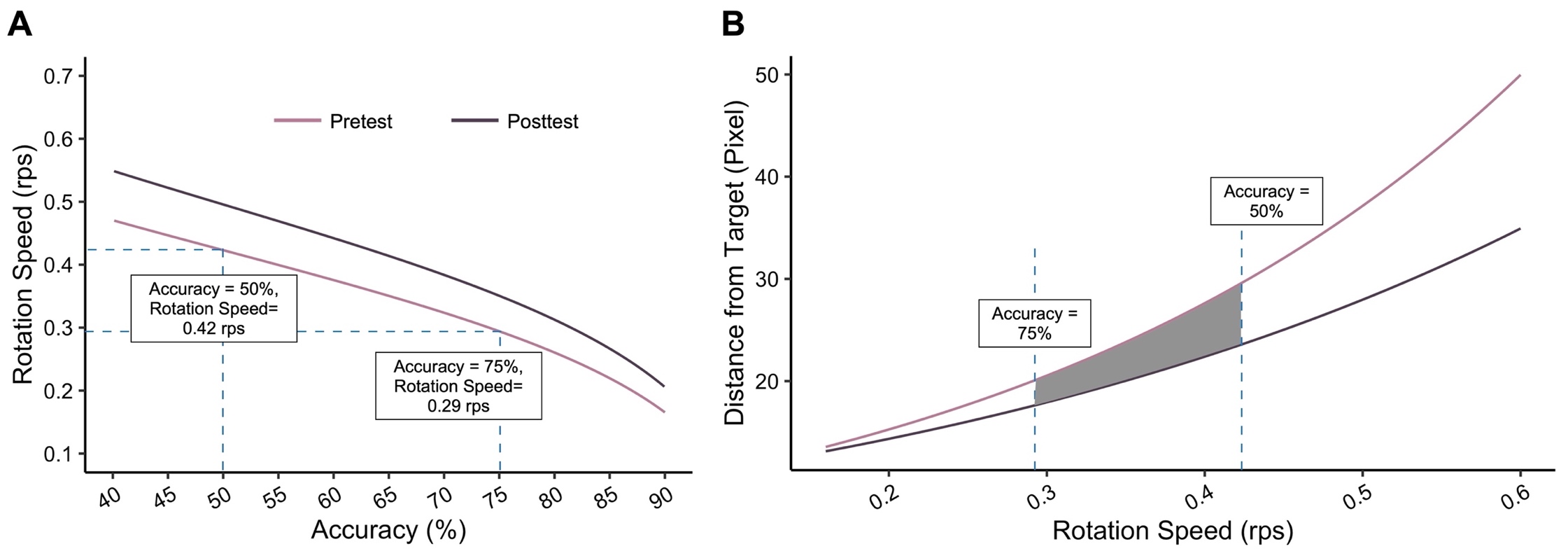


**Supplementary Figure 1.** Demonstration of the logistic curves with psychometric functions. Pretest and posttest logistic curves are illustrated by the pink and purple curves, respectively. Both **(A)** and **(B)** show that the pipeline for calculating the individual volunteer’s performance accuracy in the pursuit rotor task. **(A)** The time on target fitting curve indicates the rotation speed at which the participants could achieve the particular accuracy level. At first, the time on target scores were converted to the accuracy levels by divided by total time in a trial and then performed the logistic curve fitting by psychometric function. With the pretest fitting curve, the rotation speeds at threshold levels from 50% to 75% in pretest were selected to be the reference to estimate the continuous measurement of learning acquisition. **(B)** The curves for rotation speed - distance from target show that the distance performance in particular rotation speed in pretest and posttest. The gray field between two curves shows the area-under-curve (AUC) difference between two tests. The AUC within 50% to 75% of tracking accuracy was regarded as the indicator of visuomotor performance. For estimate the area under pretest and posttest curve, the AUC within the interval of the rotation speeds at 75% to 50% of tracking accuracy in pretest would be calculated individually in pretest and posttest curves. The AUC difference between pretest and posttest divided by the AUC of pretest was regarded as the measurement of visuomotor improvement. The best performance accuracy was estimated by the maximum time on target and minimum distance from target fitting curves, and the mean performance accuracy was estimated by the mean time on target and distance from target fitting curves.


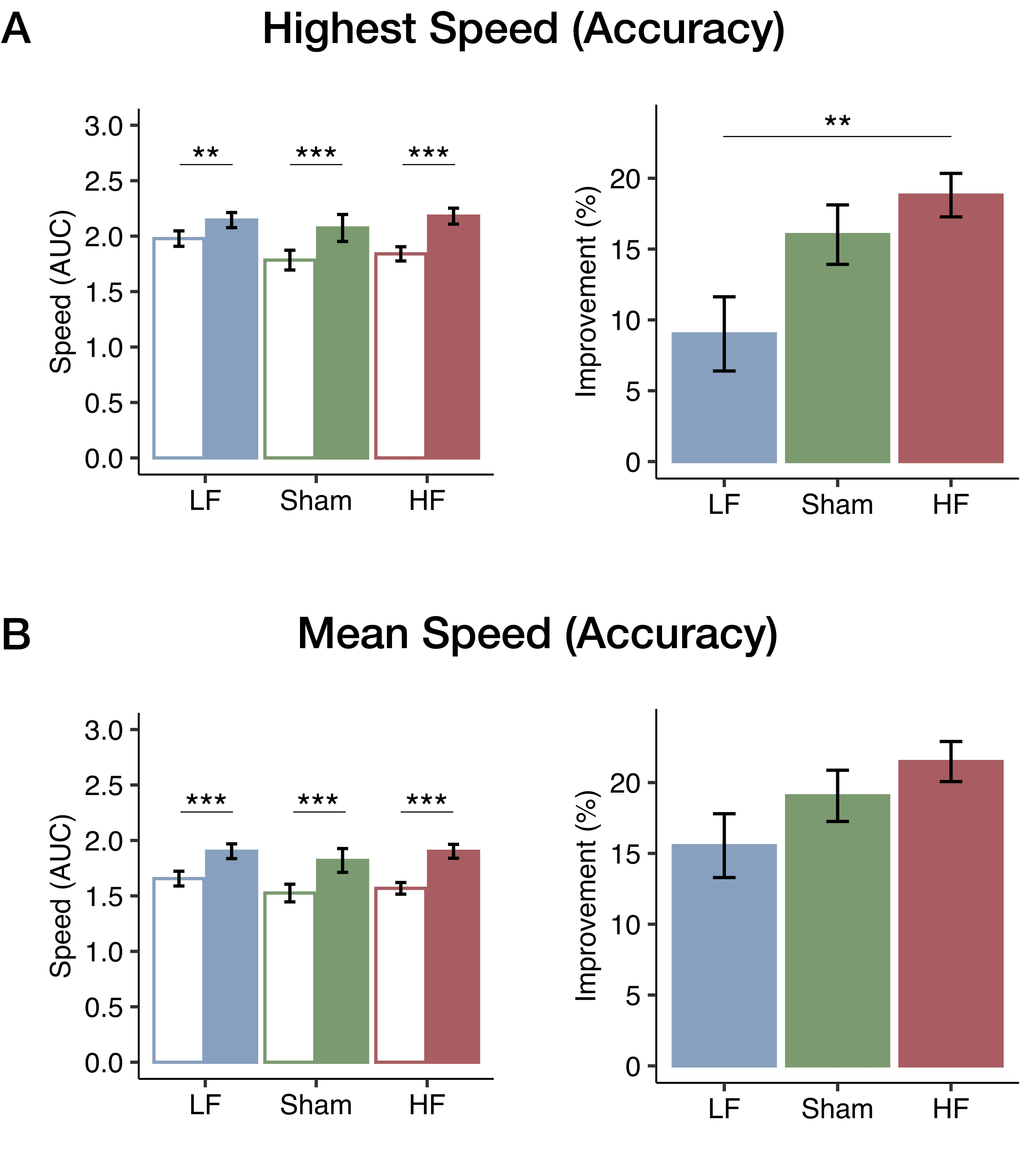


**Supplementary Figure 2.** Changes in visuomotor accuracy in the highest and mean rotation speed among the three rTMS groups. The bars (blue: LF; green: sham; red: HF) represent the mean AUC of speed (left panel) in the pretest (empty bar) and posttest (solid bar) and mean improvements (right panel). **(A)** The performance and improvements in the highest rotation speed. All groups showed significant increases in the highest speed; moreover, the HF group had not only significantly higher speed but also larger improvement than that of the LF group. **(B)** The performance and improvements in the mean rotation speed. All groups showed significant increases after intervention. Error bars indicate the standard error of the mean. The Bonferroni correction was applied to all *p*-values (**: *p* < 0.01; ***: *p* < 0.001).


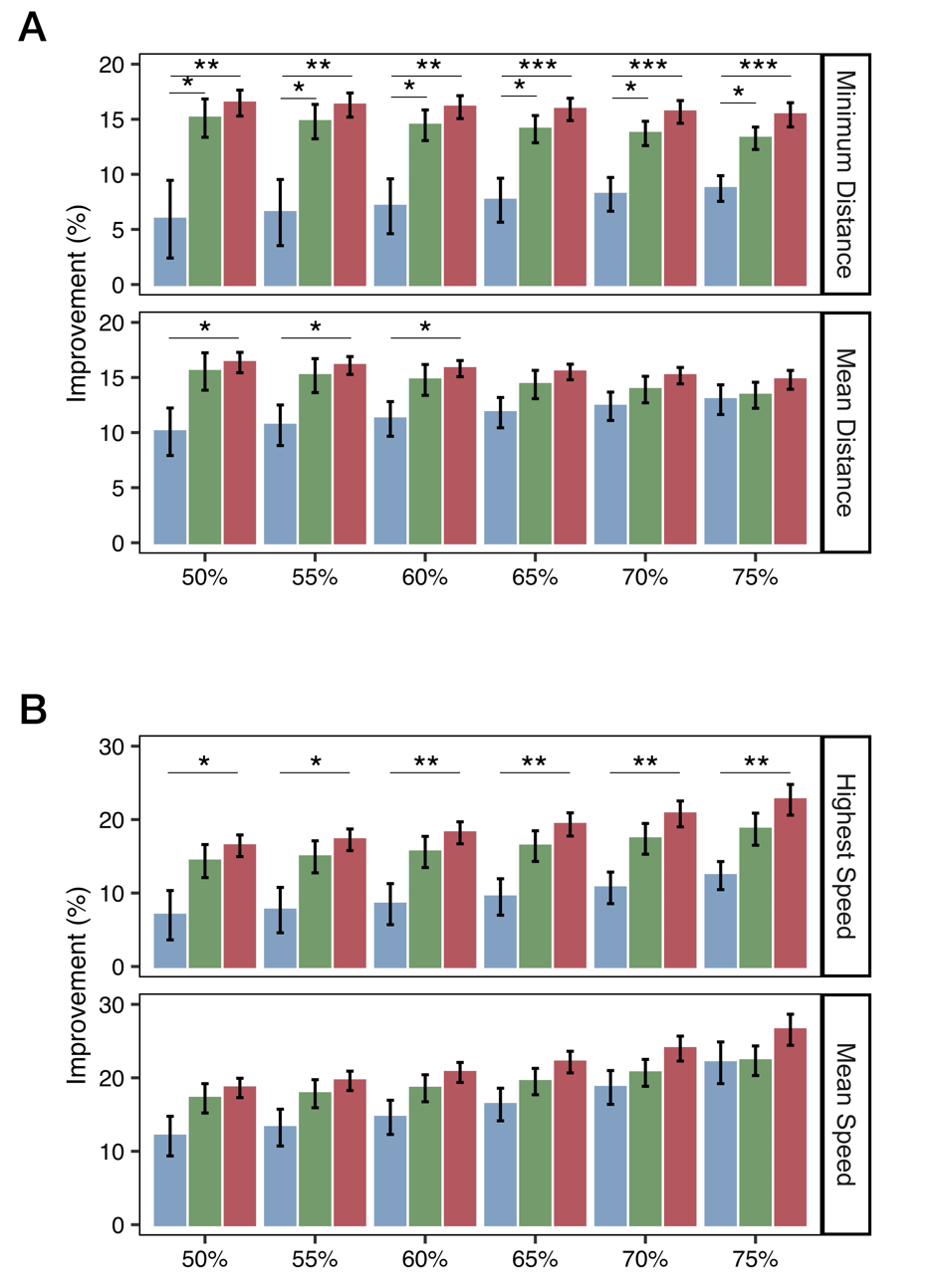


**Supplementary Figure 3.** Performance improvements in distance from target and time on target among the three rTMS groups at particular accuracy levels. The pairwise t-tests with Bonferroni correction were conducted to compare the differences among rTMS groups at each accuracy level. **(A)** The bars (blue: LF; green: sham; red: HF) represent the estimated mean improvements in the minimum (top panel) and mean (bottom panel) distance from target. In the minimum distance, the LF group showed significant lower improvements than HF and sham groups at all the accuracy levels. In the mean distance, the LF group also had significant lower improvements than the HF group at the accuracy levels from 50 to 60%. **(B)** The bars represent the estimated mean improvements in the highest (top panel) and mean (bottom panel) rotation speed. In the highest speed, the LF group had significant lower improvements than the HF group at all the accuracy levels. Error bars indicate the standard error of the mean. The Bonferroni correction was applied to all *p*-values (*: *p* < 0.05; **: *p* < 0.01; ***: *p* < 0.001).


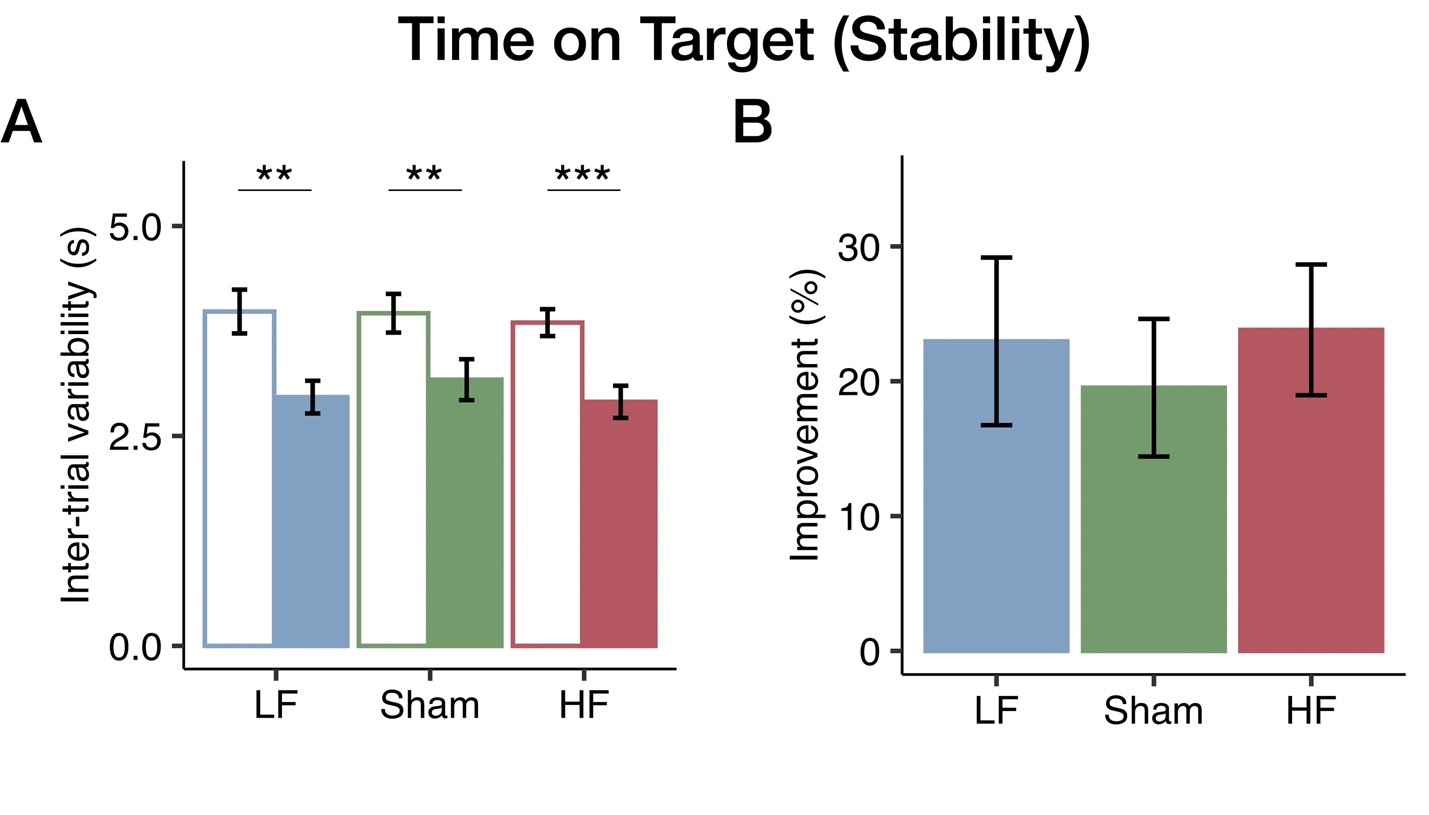
**Supplementary Figure 4.** Changes in visuomotor stability in the time on target among the three rTMS groups. **(A)** The bars (blue: LF; green: sham; red: HF) represent the mean intertrial variability in the pretest (empty bar) and posttest (solid bar). All groups had significant decreases in intertrial variability. **(B)** The bars represent the mean improvements in the intertrial variability in the time on target. Error bars indicate the standard error of the mean. The Bonferroni correction was applied to all *p*-values (**: *p* < 0.01; ***: *p* < 0.001).
